# New Polymer Inclusion Membranes in the Separation of Palladium, Zinc and Nickel Ions from Aqueous Solutions

**DOI:** 10.3390/polym13091424

**Published:** 2021-04-28

**Authors:** Elżbieta Radzyminska-Lenarcik, Ilona Pyszka, Wlodzimierz Urbaniak

**Affiliations:** 1 Faculty of Chemical Technology and Engineering, UTP University of Science and Technology, 85-796 Bydgoszcz, Poland; ilona.pyszka@utp.edu.pl; 2 Faculty of Chemistry, Adam Mickiewicz University, 61-712 Poznan, Poland; Wlodzimierz.Urbaniak@amu.edu.pl

**Keywords:** polymer inclusion membrane, alkyltriazole derivatives, palladium, zinc, nickel, solvent extraction

## Abstract

The new polymer inclusion membrane (PIM) with a 1-alkyltriazole matrix was used to separate palladium(II) ions from aqueous chloride solutions containing a mixture of Zn-Pd-Ni ions. The effective conditions for transport studies by PIMs were determined based on solvent extraction (SX) studies. Furthermore, the values of the stability constants and partition coefficients of M(II)-alkyltriazole complexes were determined. The values of both constants increase with the growing hydrophobicity of the 1-alkyltriazole molecule and have the highest values for the Pd(II) complexes. The initial fluxes, selectivity coefficients, and recovery factors values of for Pd, Zn and Ni were determined on the basis of membrane transport studies. The transport selectivity of PIMs were: Pd(II) > Zn(II) > Ni(II). The initial metal ion fluxes for all the cations increased with the elongation of the alkyl chain in the 1-alkyltriazole, but the selectivity coefficients decreased. The highest values of the initial fluxes at pH = 4.0 were found for Pd(II) ions. The best selectivity coefficients Pd(II)/Zn(II) and Pd(II)/Ni(II) equal to 4.0 and 13.4, respectively, were found for 1-pentyl-triazole. It was shown that the microstructure of the polymer membrane surface influences the kinetics of metal ion transport. Based on the conducted research, it was shown that the new PIMs with 1-alkyltriazole can be successfully used in an acidic medium to separate a mixture containing Pd(II), Zn(II) and Ni(II) ions.

## 1. Introduction

Polymer inclusion membranes (PIMs) are more and more often used due to both the environmental benefits and the efficiency of separation of valuable metals from various solutions, including sewage. Membranes of this type consist of a metal carrier, a plasticizer (e.g., 2-nitro phenyl octyl ether, 2-nitro phenyl pentyl ether) and a polymer matrix (e.g., cellulose triacetate or PVC).

Palladium, zinc and nickel are widely used in various fields. For example, these metals are used as catalysts for chemical reactions [1,2,3] and hydrogen production [4]. Their limited sources and growing industrial demand should support the development of methods of their recovery. Low content, palladium and nickel especially, and the complex composition of the waste require special separation methods [5].

The technological schemes currently in use employ different enrichment variants such as ionic flotation [6], magnetic or electrical separation [7,8], extraction [9,10,11], adsorption [12], biosorption [13,14], phytoremediation [15] or ion exchange [16,17,18]. Each of these methods makes it possible to obtain concentrates that, due to the value of the extracted metallic elements, are most often processed by hydrometallurgical methods [19,20] based on leaching and extraction. Quaternary phosphonium salts in the presence of toluene [21], amide derivatives [9], pyridine ammonium derivatives [10] and ionic liquids [22] are used as selective palladium extractants. What is more, 1,2,4-triazoles and their amino derivatives have also been studied as extractants of Co(II), Ni(II), Zn(II) and Cd(II) ions [23]. Moreover, the effectiveness of removing Cu(II) [24] and Ni(II) [25] ions during SX with 1-alkyl-1,2,4-triazoles has been investigated.

Triazoles are heterocyclic compounds containing three nitrogen atoms in a five-membered ring. They are known primarily as compounds that exhibit biological activity. For example, they have antibacterial, antihistamine, analgesic, insecticidal, antifungal, and antitumor properties [26,27]. Triazoles are used as catalyst components [28,29,30] and anticorrosive agents [31,32]. They are also applied in fluorescent material production [33] and plant protection products [26,34,35,36].

Based on the results of research on alkylimidazoles [37,38,39,40,41,42,43,44] and the search for new effective extractants of Pd(II) ions from the acidic environment, it was assumed that alkyltriazoles can affect the efficiency of Pd(II) separation as well as other precious metals (Pt, Au, Ag).

This work aims to investigate the usefulness of 1-alkyltriazoles (alkyl–from pentyl up to hexadecyl) in the separation of Pd(II) from a mixture of Zn(II), Ni(II) and Pd(II) ions and evaluate the suitability of alkyl triazole derivatives in the separation of palladium from a mixture. The solvent extraction (SX) and the transport of mixture Pd-Zn-Ni ions across PIMs with 1-alkyltriazoles were investigated to achieve this. The values of the stability constants and the partition coefficients were determined using extraction data. The values of initial flux (J_0_) and selectivity coefficient (S_M(1)/M(2)_) of a given metal after 24 h were selected for the comparative analysis of the transport process.

## 2. Materials and Methods

### 2.1. Reagents and Equipment

Inorganic chemicals: potassium, zinc(II), nickel(II) and palladium(II) chlorides, hydrochloric acid (HCl), potassium hydroxide (KOH) were of analytical grade, and were purchased from POCh (POCh, Gliwice, Poland). Aqueous solutions were prepared with double distilled water (conductivity 0.1 µS/m). The initial concentration of Pd(II), Zn(II) and Ni(II) was determined by titration with EDTA (POCh, Gliwice, Poland). The potassium chloride concentration was determined gravimetrically as sulfate.

Alkyl triazole derivatives **1**–**9** (Table 1) were synthesized by prof. A. Skrzypczak (Poznan University of Technology, Poznan, Poland) in the alkylation reaction of 1,2,4-triazole according to Equation (1).

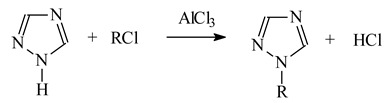
(1)

Physical properties of the 1-alkyl-1,2,4-triazoles (**1**–**9**) are collected in the Table 1.

Cellulose triacetate (CTA) (CTA, above 98%, Sigma-Aldrich, Poznan, Poland), *o*-nitrophenyl pentyl ether (*o*-NPPE, Fluka, Busch, Switzerland), dichloromethane (Fluka, Busch, Switzerland) and tetramethylammonium hydroxide (POCh, Gliwice, Poland) were also used.

The pH-meter (PHM 250 (Radiometer, Copenhagen, Denmark) equipped with electrode C 2401-8 (Radiometer, Copenhagen, Denmark) and AAS 240FS Spectrometer, Agilent, Santa Clara, CA, USA (AAS-atomic absorption spectroscopy) were used to measure the pH value and the metal ions concentrations, respectively. Measurements were made for the following emission lines of the analyzed elements Pd 247.6 nm, Zn 213.9 nm and Ni 232.0 nm.

### 2.2. Determination of Dissociation Constants (pK_a_)

The dissociation constants (pK_a_) of alkyl triazole derivatives (**1**–**9**) was determined by potentiometric titration. The weighted sample of alkyl triazole derivatives were dissolved in a solution containing hydrochloric acid with ionic strength I = 0.5 in excess. Next, this solution was titrated with a standardized KOH solution corrected for I = 0.5 with KCl.

### 2.3. Liquid–Liquid Extraction Procedure (SX)

The measurements were carried out at 20 °C and at a fixed ionic strength (I = 0.5 mol/dm^3^ KCl). The initial concentrations of metal ions and HCl in the aqueous phase were constant and were 0.01 and 0.1 mol/dm^3^, respectively. The ligand (1-alkyl-triazoles **1**–**9**) concentration in methylene chloride was varied from 0.01 to 0.03 mol/dm^3^. Equal volumes of organic and aqueous phases were mechanically shaken for 25 min. After establishing the equilibrium, the phases were separated. Equilibrium pH of aqueous phases and metal concentrations were measured.

### 2.4. Polymer Inclusion Membrane

Polymer inclusion membranes were prepared as reported in the earlier paper [34,35,36,37,40,45]. The membrane contained 2.7 cm^3^ o-NPPE/1 g CTA, and 0.5–1.5 mol/dm^3^ of 1-alkyltriazole **1**–**9** (Table 1) based on a plasticizer.

The thickness of the PIM was measured using a digital micrometer (Panametrics^®^ Magna-Mike^®^ 8500 (San Diego, CA, USA)) with an accuracy of 0.1 µm. A surface characterization study of the polymer inclusion membranes was performed by atomic force microscopy (AFM) using Atomic-force MultiMode Scanning Probe Microscope IIIa (Digital Instruments Vecco Metrology Group, Santa Barbara, CA, USA). The analysis of surface pore characteristics of the polymer membrane was made using the NanoScope v.5.12 AFM image processing program, which enabled the calculation of roughness (R_q_).

### 2.5. Transport Studies

Transport experiments were carried out in the system described in earlier papers [34,35,36,37,40,45] at 20 ± 0.2 °C. The feed phase was an aqueous solution of metal ions with a concentration of C_0,M_ = 0.001 mol/dm^3^ each. The feed phase pH was kept constant (pH = 4.0). The receiving phase was 0.01 mol/dm^3^ HCl. At the receiving phase, metal ions concentrations were measured.

## 3. Results and Discussion

### 3.1. Determination of Dissociation Constants (pK_a_)

Alkyl-triazole derivatives (**1**–**9**) (L) dissociate according to the reaction:

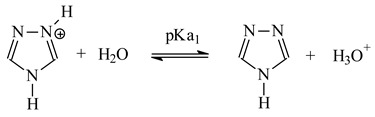
(2)

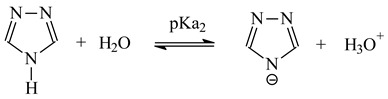
(3)

The equilibrium constant (pK_a_) of protonated form of alkyltriazoles (HL) is defined:

(4) $$K_{a_{1}}=\frac{\left[L\right]\left[H_{3}O^{+}\right]}{\left[HL^{+}\right]}\;K_{a_{2}}=\frac{\left[L^{-}\right]\left[H_{3}O^{+}\right]}{\left[L\right]}$$

where K_a,1_ and K_a,2_ is the dissociation constant of the protonated ligand (HL^+^) and ligand (L), [HL^+^] is the concentration of the conjugate acid of the ligand equal to the analytical concentration of HCl (mol/dm^3^) in the aqueous phase and [L] is the concentration of the free ligand in the aqueous phase.

The pK_a_ values, determined by potentiometric method (methodology in Section 2.2), are presented in the Table 2 together with pK_a_ values for 1,2,4-triazole.

Both the 1,2,4-triazole and 1-alkyl-1,2,4-triazoles (**1**–**9**) in aqueous solutions are very weak bases (Table 2). Their alkalinity is five orders of magnitude lower than that of ammonia and 1-alkylimidazoles (azoles with two nitrogen atoms in the ring). According to Pearson’s theory of hard and soft acids and bases (HSAB), 1-alkyltriazoles are soft bases.

### 3.2. SX of Metal Ions by 1-Alkyl-1,2,4-Triazole (**1**–**9**)

In order to understand the transport of metal ions across the PIMs doped with alkyl-triazole **1**–**9** as a better ion carrier, it was necessary to perform solvent extraction studies with alkyl-triazole **1**–**9** as an extractant. Solvent extraction was performed from solutions containing single Ni(II), Zn(II) and Pd(II) ions.

Based on measurements of metal concentrations in the aqueous phase after partition, equilibrium is achieved; in case there was no phase change in volume (V_aq_ = V_org_), the distribution ratio (D_M_) of the metal ion was calculated using the following formula:

(5) $$D_{M\;}=\frac{C_{M\left(org\right)}}{C_{\left(aq\right)}}=\frac{C_{0,M}-C_{M}}{C_{M}}$$

where: C_0,M_ and C_M_ denote analytical metal ion concentrations in the aqueous phase before and after attaining partition equilibrium, respectively.

**Figure 1:**
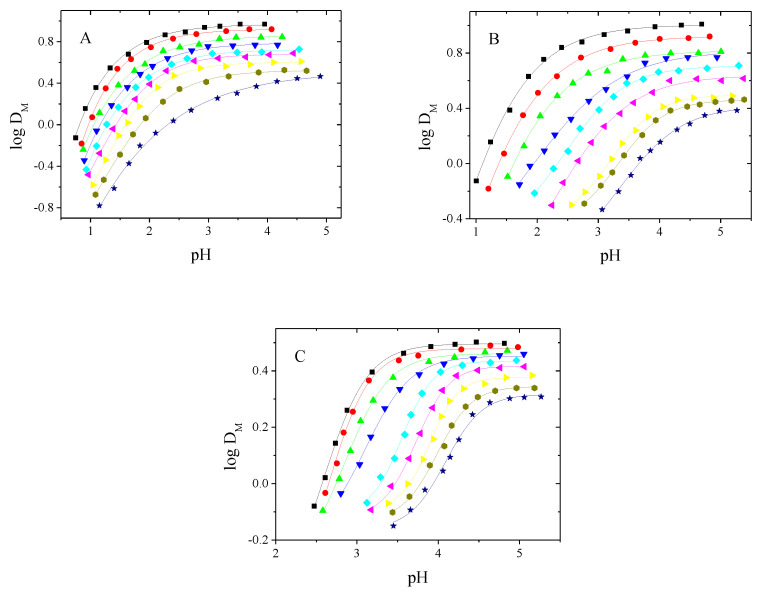
Extraction curves (log D_M_ = f (pH)) of Pd(II) (**A**), Zn(II) (**B**) and Ni(II) (**C**) complexes. ★-**1**, 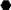-**2**, ⏵-**3**, ⏴-**4**, ♦-**5**, ▼-**6**, ▲-**7**, ●-**8**, ■-**9**.

Figure 1 shows the extraction curves of the partition of the complexes of tested metals with 1-alkyltriazol between the aqueous and organic phases.

Figure 1 shows that the distribution ratio of each metal ion complex increases with the increasing pH of the aqueous phase. According to the D_M_ values, the extraction efficiency depends on the type of metal ion and increase in order Pd(II) > Zn(II) > Ni(II).

The pH_1/2_ values corresponding to 50% metal extraction for Pd, Zn and Ni are collected in the Table 3.

It is evident from the data in Table 3 that the extraction process proceeds in an acidic medium, and the pH_1/2_ value decreases with increasing alkyl chain length at position 1 of the 1-alkyltriazole moiety and varies between pH ranges of 0.80–2.30, 1.10–3.60 and 2.57–3.90 for Pd(II), Zn(II) and Ni(II) complexes, respectively.

The percentage extraction of each metal ion calculated using formula:

(6) $$\%E=\frac{D_{M}\cdot 100\%}{D_{M}+V_{aq}/V_{org}}$$

The highest percentage extraction of palladium (89%), zinc (74%), and nickel (42%) obtained for 1-hexadecyl-triazole (**9**).

### 3.3. Determination of the Equilibrium Constants of SX

On the basis on SX studies by a liquid–liquid partition method, both the partition constants (P_n_) and the stability constants (β_n_) of Pd(II), Ni(II) and Zn(II) complexes with 1-alkyl-1,2,4-triazoles (**1**–**9**) were determined. This method was previously used to determine the extraction constants of Cu(II) [41,42,43], Co(II) [44,46], Ni [47] and Zn [48,49] complexes with alkyl-imidazoles and Zn(II), Ni(II), Cu(II), Co(II) and Cd(II) with ethylenodiamino-bis-acetylacetone [45]. A detailed method of determining the extraction constants is described in the papers [41,42,43,44,46,47,48,49].

On the bases pH measurements, allowing us to determine the equilibrium concentration of free lignd (alkyltriazole) [L], the values of the extraction constants were calculated on the basis of the modified Rydberg formula:

(7) $$D_{M}=\frac{P_{n}\beta_{n}{\left[L\right]}^{n}+P_{n+1}\beta_{n+1}{\left[L\right]}^{n+1}+\ldots +P_{N}\beta_{N}{\left[L\right]}^{N}}{\sum_{n=0}^{n=N}\beta_{n}{\left[L\right]}^{n}}$$

where *n* is the number of ligand particles in the first metal ion complex which is hydrophobic to the extent that it is possible for it pass freely into the organic phase [50,51].

The extraction constants of M(II)–1-alkyltriazole complexes, determined on the basis of the Equation (7), are collected in Table 4 together with the stability constants, previously determined for Ni(II) and Zn(II) complexes with 1,2,4-triazole [23].

The data in Table 4 show that the stability constant of Pd(II), Zn(II) and Ni(II) complexes with 1-alkyl-1,2,4-triazoles depend on the length of the alkyl chain and they increase with an increase in its length (with an increase in hydrophobicity). In the same way, the partition constants of these complexes change. The stability of the complex also depends on the properties of the central ion. For complexes with 1-alkyl-1,2,4-triazoles, it decreases respectively: Pd > Zn > Ni.

The Pd(II) ions form 4-coordination square planar complexes [PdL_4_]^2+^. In the case of Zn(II), an additional phenomenon is the ease of changing coordination number from 6 to 4 [45,48,49,52]. This is illustrated by Equation (8).

[Zn(H_2_O)_6_]^2+^ + *n*L ↔ [Zn(H_2_O)_4-*n*_L*_n_*]^2+^ + (*n* + 2)H_2_O (8)

The Ni(II) ions form 6-coordination complexes [NiL_6_]^2+^, which have a rigid octahedral structure. The formation of tetrahedral or square planar complexes enhances the extraction of Zn(II) and Pd(II).

### 3.4. Transport of Pd(II), Zn(II) and Ni(II) Ions across Polymer Inclusion Membranes (PIMs)

#### 3.4.1. The Concentration of Carrier

In preliminary experiments, no metal ion transport across a membrane containing only the support (CTA) and plasticizer (*o*-NPPE), i.e., in the absence of an ion carrier, was observed. For the blank experiment, no transport was detected for more than 24 h of the continuous process run.

Then, the influence of the carrier concentration in the membrane on separation efficiency of the Pd(II), Zn(II) and Ni(II) ions from their equimolar mixture was determined. According to Danesi [53], if the relationship ln(C_0,M_ − C_M_)/ = f(t) as a function of time shows a high linear correlation, the kinetics of metal ion transport across membranes was described by the following Equation:

(9) $$\frac{\ln\left(C_{0,\;M}-C_{M}\right)}{C_{M}}=-kt$$

where c_M_ and c_0,M_ are the metal ion concentrations (mol/dm^3^) in the feed phase at a given time, and the initial metal ion concentrations, respectively; k is the first order rate constant (s^−1^) and t is the time of transport (s) [53,54].

The value of the rate constant (k) of the metal ions transport was determined from the angle of the straight line ln(C_0,M_ − C_M_)/ = f(t).

According to Danesi [53], the permeability coefficient (P, m/s) of metal ions across the membranes was described by the following equation:

(10) $$P=-\frac{V}{A}\;k$$

where: V is the volume of the aqueous source phase (m^3^), and A is an effective membrane area (m^2^).

The initial flux (J_0_) was calculated as being equal to:

(11) $$J_{0}=P\cdot c_{0}$$

The values of initial fluxes for competitive transport of Pd(II), Zn(II) and Ni(II) across PIMs vs. concentration of 1-pentyl-1,2,4-triazole (**1**) in membrane are shown in Table 5 together with the values of the metal recovery factor, calculated from the Formula (12):

(12) $$RF=\frac{C_{0,M}-C}{C_{0,M}}\cdot 100\%$$

The fluxes of all the metal ions rapidly increase with the increase of carrier concentration in the membrane up to a 0.5 mol/dm^3^ concentration calculation on the plasticizer’s volume (Table 5). The highest initial fluxes of Pd(II) are found at the 0.5 mol/dm^3^ concentration. Above this concentration, the rate of Pd(II) ion transport is slightly lower but for the remaining two cations the initial flux increases, especially for Zn(II), which can form two types of complexes at higher carrier (ligand) concentrations (Equation (13)).

[Zn(H_2_O)_6_]^2+^ + 4L + 2A^−^ ↔ [Zn(H_2_O)_2_L_4_]A_2_ + 4H_2_O [Zn(H_2_O)_6_]^2+^ + 4L + 2A^−^ ↔ [ZnL_4_]A_2_ + 6H_2_O (13)

Optimal membranes for the separation of palladium from the Pd-Zn-Ni mixture consisting of NPPE/1g CTA, and 0.5 mol/dm^3^ of 1-alkyl-1,2,4-triazole (**1**–**9**).

#### 3.4.2. Membrane Characterization

The thickness of membranes before and after transport was found to be the same. The average PIM thickness for investigated membranes are collected in the Table 6.

**Figure 2:**
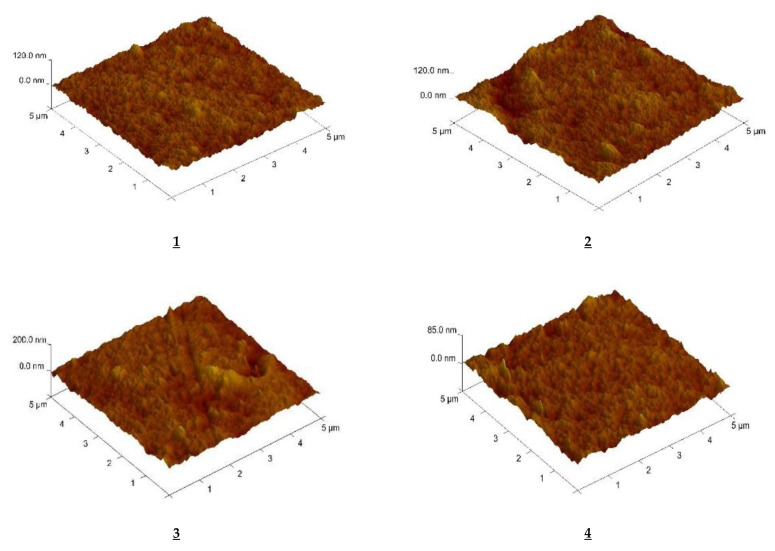

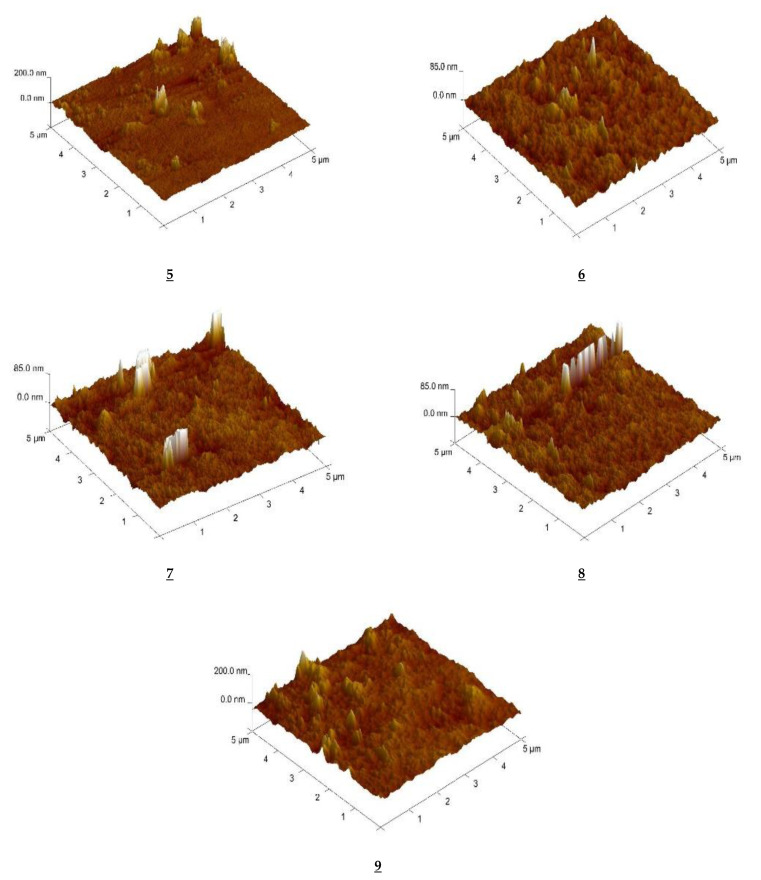
AFM pictures of the PIMs with 1-alkyl-triazole **1**–**9**.

Figure 2 shows an AFM image of PIM’s with 1-alkyltriazole as the carrier in three-dimensional form with format of 5.0 × 5.0 µm^2^. The distribution of the carrier in the investigated membrane after evaporation of the methylene chloride is homogeneous.

**Figure 3:**
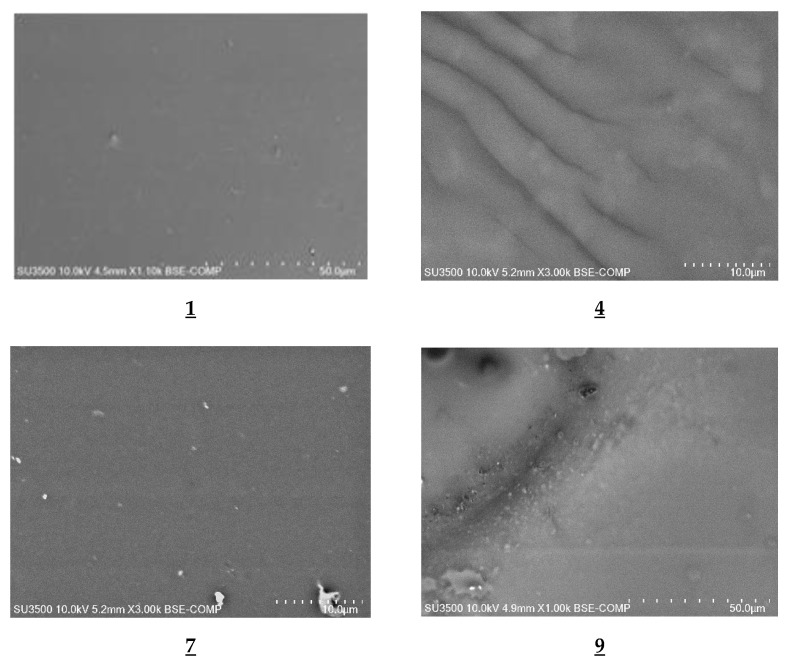
SEM images of PIMs containing 1-alkyl-triazole **1**,**4**,**7**,**9** as a carrier.

The SEM images (Figure 3) showed that all membranes had dense and homogeneous structures. The carrier’s molecules could have crystallized in the membrane and have migrated to the membrane surface, causing its roughness and porosity.

The roughness (R_q_) parameter of the membrane is the standard deviation of the z values within the box cursor and was calculated using atomic force microscopy (AFM) from Formula (14) and is presented in Table 6.

(14) $$R_{q}=\sqrt{\frac{\sum {\left(z_{i}\right)}^{2}}{n}}$$

In the formula given above, z_i_ is the current z value, *n* is the number of points within the box cursors.

From the data in Table 6, the roughness of the membranes increases with the increasing length of the alkyl substituent in the carrier molecule.

The roughness values determined for membranes with carriers **2**–**9** are insignificant and higher than for the commercial D2EHPA carrier (4.7 nm) used by Salazar-Alvarez [55].

Roughness values for CTA-o-NPPE-triazole membranes are comparable both with those found in PIMs with alkylimidazole (3.7–7.2 nm) [56] and with an azothiacrown ethers containing imidazole molecules in the ring (3.3–5.3 nm) [57].

#### 3.4.3. The Concentration of Chloride Ions in the Feed Phase

The influence of the concentration of chloride ions in the feed phase on the values of initial fluxes of the investigated metal ions was also examined. Table 7 shows the values of the initial fluxes (J_0_) as a function of the concentration of chloride ions in the feed phase together with the values of the palladium ion separation coefficients (S_M1/M2_) in relation to the remaining ions, which were calculated from the following Formula:

(15) $$S_{M_{1}/M_{2}}=\frac{J_{0,M_{1}}}{J_{0,M_{2}}}$$

Increasing the concentration of chloride ions in the feed phase facilitates the formation of chloride complexes of the metals studied, 4-coordinated in the case of Pd(II) and Zn(II) and 6-coordinated for Ni(II) (Equation (16)).

for Pd(II) and Zn(II)  [M(H_2_O)_6_]^2+^ + 4Cl^‾^ → [MCl_4_(H_2_O)_2_]^2−^ + 4H_2_O for Ni(II)    [M(H_2_O)_6_]^2+^ + 6Cl^‾^ → [MCl_6_]^4−^ + 6H_2_O (16)

While Table 7 shows that increasing the concentration of chloride ions in the feed phase increases the initial flux values of all metal ions, especially Pd(II) and Zn(II) ions, which can form 4-coordinate complexes. The phenomenon increases the J_0_ values and, at the same time, reduces the selectivity coefficients of Pd(II)/Zn(II) and Pd(II)/Ni(II).

**Figure 4:**
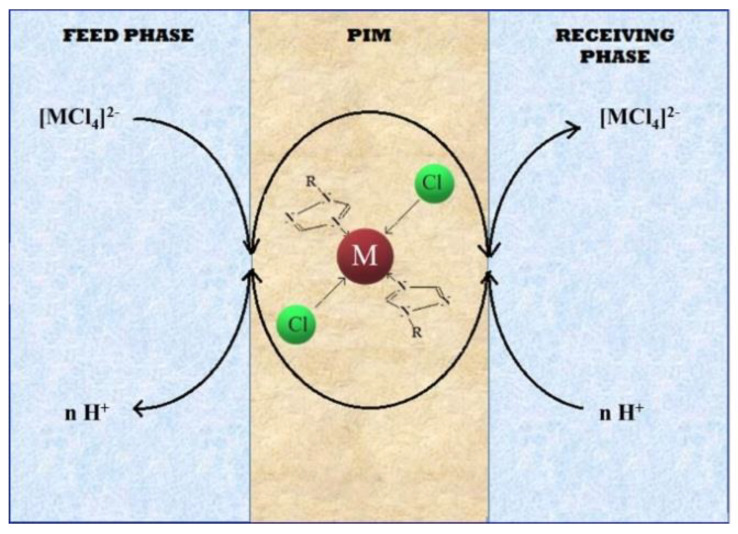
Transport scheme across PIMs with 1-alkyl-triazole (**1**–**9**).

The process of transport of metal ions across PIMs with 1-allyltriazoles as carriers is shown in Figure 4.

### 3.5. Metal Ions Transport across PIMs

In the next experiment, the transport of Pd(II), Zn(II), and Ni(II) ions into the receiving hydrochloric acid across a PIMs with 1-alkyl-triazole **1**–**9** was carried out. The experimental results are summarized in Table 8.

As shown by the results presented in Table 8, the initial fluxes of metal ions transported across PIMs with 1-alkyl-1,2,4-triazole for all the cations increase with elongation of the alkyl substituents in the carriers’ molecules, but the selectivity coefficients decreased in the same direction. Moreover, the initial fluxes of metal ions increase both with an increase in the basicity of the carrier molecules (1-alkyltriazole) (Table 2) and with an increase in stability constants of the Pd(II), Zn(II) and Ni(II) complexes (Table 4).

The initial fluxes decrease in the following order: Pd(II) > Zn(II) > Ni(II). In the case of Pd(II), the higher initial flux values may be due to the formation of planar square complexes with 1-alkyltriazole (**1**–**9**) as well as higher values of their stability constants.

The highest separation coefficients (S) Pd(II)/Zn(II) and Pd(II)/Ni(II) were found for 1-pentyl-triazole (**1**). They are 4.0 and 13.4 for Pd/Zn and Pd/Ni, respectively.

### 3.6. Diffusion Coefficients

**Figure 5:**
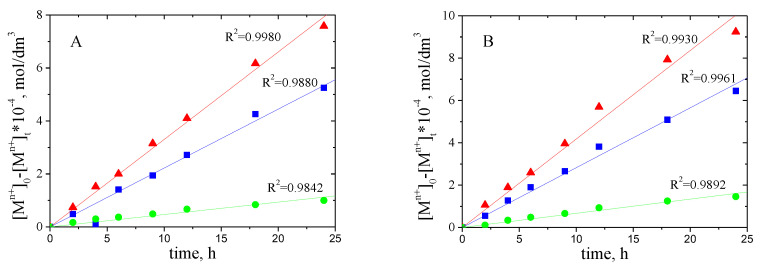
Dependence [M^2+^]_0-_[M^2+^]_t_ vs. time for ▲-Pd (II), ■-Zn (II), and ●-Ni (II) ions transport across PIMs with 1-pentyl-triazole (**A**) and 1-hexadecyl-triazole (**B**).

In Figure 5, the correlation graphs [M^2+^]_0-_[M^2+^]_t_ versus time of Pd(II), Zn(II) and Ni(II) ions transport across PIM with 1-pentyl-triazole (**1**) and 1-hexadecyl-triazole (**9**) is presented.

The diffusion coefficient of Pd(II), Zn(II) and Ni(II) (D_o_)was calculated from the equation:

D_o_ = d_o_/Δ_o_ (17)

where: d_o_ is the thickness of the membrane (Table 6) and Δ_o_ could be evaluated by plotting [M^2+^]_0-_[M^2+^]_t_ vs. time (Figure 5).

The corrected (normalized) membrane diffusion coefficient (D_o,n_), which considers the morphological features inside the membrane (ε-porosity and τ-tortuosity), was calculated from the equation described by Salazar-Alvarez et al. [55]:

D_o,n_ = D_o_∙(ε/τ) (18)

The porosity (ε) of the membrane was calculated using atomic force microscopy (AFM). The membrane tortuosity was determined from the relationship developed by Wolf and Strieder [58]:

(19) τ=1−lnε

Obtained values of diffusion coefficients are presented in Table 9.

On the basis of their values, it can be concluded that the limiting stage of transport is the process of transferring the metal complex to the membrane.

The values of diffusion coefficient determined in this study are in the range of 10^−7^ to 10^−11^ cm^2^/s (Table 9). They are comparable with those given in literature (10^−12^ to 10^−6^ cm^2^/s). On the basis of their values can be concluded that the limiting step of transport is the transferring of metal complex across membrane. The value of the diffusion coefficient of M(II)- 1-alkyl-triazole complexes is smaller than the value for the Pb(II) complex with (D2EHPA (D_0_ = 1.5 × 10^−7^ cm^2^/s) in PIM’s reported by Salazar-Alvarez et al. [53] and comparable to PIMs with 1-alkylimidazole for which the values of diffusion coefficients range from 10^−12^ to 10^−8^ cm^2^/s [54].

The values of normalized diffusion coefficients of M(II) alkyltriazole complexes, obtained in transport across PIM’s containing 1-alkyl-triazole (**1**,**9**) are in the range of 4.83 × 10^−12^ to 4.53 × 10^−8^ cm^2^/s.

### 3.7. Recovery of Metal

**Figure 6:**
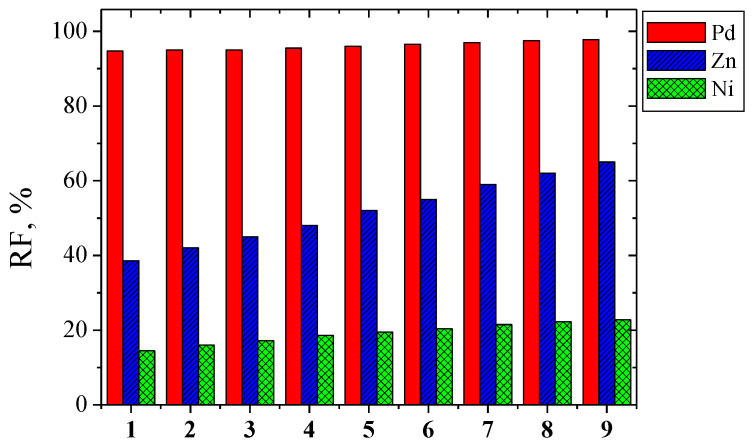
Recovery factor values of Pd(II), Zn(II), and Ni(II) vs. time transport (after 24 h) across PIMs with 1-alkyl-triazoles **1**–**9**.

From the separation point of view, the most important is a complete transport of the solute to the receiving phase, described by recovery factor (RF). It was calculated from Equation (11). Figure 6 shows the values of the recovery factor Pd(II), Zn(II), and Ni(II) ions from the feed phase during the 24-hrs transport across PIMs with 1-alkyl-triazoles.

The data in Figure 5 show that the Pd(II) recovery factors are the highest and amount to 94–98% for all the tested membranes. For Zn(II) ions, the values of RF strongly depended on the type of carrier and changes with increasing hydrophobicity of the carrier molecule from 39 to 65% In the case of Ni(II) ions, the increase in the hydrophobicity of the carrier molecule had a slight effect on the RF values (RF varies from 14.5% to 23%).

The obtained results were compared with the literature data [59,60].

Mohdee et al. [59] investigated the separation of Pd(II) from wastewater containing Pd(II)-Cu(II)-Ni(II) using a liquid membrane on a hollow fiber carrier from Aliquat 336 at pH 2 feed solution. They applied 0.5 mol/dm^3^ thiourea mixed with 0.1 mol/dm^3^ HCl as the receiving phase. Pd(II) extraction and stripping reaches over 99% and 87.09%, respectively.

For the separation of Pd(II) Regel-Rosocka et al. [60] used CTA membranes containing phosphonium ionic liquids as metal ion carriers. Pd(II) was most effectively recovered using Cyphos IL 102 and 3 mol/dm^3^ HCl as the receiving phase (48% Pd recovered) and Cyphos IL 101 and a mixture of 0.1 mol/dm^3^ thiourea + 0.5 mol/dm^3^ HCl (more than 60% Pd recovered).

The efficiency of Pd(II) separation using the CTA alkyltriazole membranes is comparable to the Aliquat 336 hollow fiber carrier fluid membrane, but better than that of CTA membranes containing phosphonium ionic liquids.

## 4. Conclusions

Palladium(II) ions can be effectively separated from equimolar aqueous solutions of Pd(II)-Zn(II)-Ni(II) chlorides using transport across polymer inclusion membranes with 1-alkyltriazoles (alkyl = pentyl, hexyl, heptyl, octyl, nonyl, decyl, dodecyl, tetradecyl, hexadecyl) as well solvent extraction in the water-methylene chloride system. The initial fluxes of each metal ion increase with an increase in the basicity of carrier (1-alkyltriazole) molecules, and also they increase in the values of the stability constants of the Pd(II), Zn(II), and Ni(II) complexes. The highest initial fluxes of metal ions were found for PIMs with 1-hexadecyltriazole, whereas for 1-pentyltriazole, the best Pd(II)/Zn(II) and Pd(II)/Ni(II) selectivity coefficients equal to 4.0 and 13.4, respectively, were found.

Increasing the concentration of chloride ions in the feed phase increases the initial fluxes of all metal ions, especially Pd(II) and Zn(II) ions.

The structure of the membranes, its roughness, and the effective pore size influence the transport process. The best membranes for Pd(II)-Zn(II)-Ni(II) separation are PIMs with 1-hexadecyltriazole. For this carrier, recovery factors of Pd(II), Zn(II), and Ni(II)ions were the highest at 98%, 65% and 23%, respectively. However, the Pd(II)/Zn(II) and Pd(II)/Ni(II) separation coefficient is the lowest.

The use of 1-alkyltriazoles as carriers in PIMs allows the process to be carried out at low pH values. It is advantageous as hydrolysis is avoided under these conditions. Moreover, 1-alkyltriazoles as soft ligands (according to HSAB theory) could enable the separation of soft cations (e.g., Pt, Pd) from hard ones, e.g., Co, Zn, Ni.

## Figures and Tables

**Table 1:** Properties of 1-alkyl-1,2,4-triazoles used in the studies.

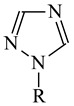	**No.**	**R**	**Compound**	**Boiling Point**, °C, **at Pressure 1 hPa**	**Melting Point**, °C
	**1**	-C_5_H_11_	1-pentyl-1,2,4-triazole	173–175	
	**2**	-C_6_H_13_	1-hexyl-1,2,4-triazole	179–181	
	**3**	-C_7_H_15_	1-heptyl-1,2,4-triazole	182–184	
	**4**	-C_8_H_17_	1-octyl-1,2,4-triazole	185–188	
	**5**	-C_9_H_19_	1-nonyl-1,2,4-triazole	201–203	
	**6**	-C_10_H_21_	1-decyl-1,2,4-triazole	216–218	
	**7**	-C_12_H_25_	1-dodecyl-1,2,4-triazole	239–241	
	**8**	-C_14_H_29_	1-tetradecyl-1,2,4-triazole	-	48–49
	**9**	-C_16_H_33_	1-hexadecyl-1,2,4-triazole	-	52–53

**Table 2:** The pK_a_ values of 1,2,4-triazole and its alkyl derivatives.

Ligand	No.	pK_a,1_	pK_a,2_
1,2,4-triazole [20]		2.5	9.89
1-pentyl-1,2,4-triazole	**1**	2.65	9.89
1-hexyl-1,2,4-triazole	**2**	2.68	9.94
1-heptyl-1,2,4-triazole	**3**	2.7	9.98
1-octyl-1,2,4-triazole	**4**	2.72	10.05
1-nonyl-1,2,4-triazole	**5**	2.73	10.06
1-decyl-1,2,4-triazole	**6**	2.75	10.06
1-dodecyl-1,2,4-triazole	**7**	2.78	10.08
1-tetradecyl-1,2,4-triazole	**8**	2.81	10.1
1-hexacedyl-1,2,4-triazole	**9**	2.85	10.14

**Table 3:** The pH_1/2_ values for extraction of Pd(II), Zn(II) and Ni(II) complexes with 1-alkyl-1,2,4-triazole.

Ligand	Metal Ions	pH_1/2_	E_max_, %
**1**	Pd(II)	2.30	67
	Zn(II)	3.60	52
	Ni(II)	3.90	14
**2**	Pd(II)	1.83	68
	Zn(II)	3.34	53
	Ni(II)	3.75	18
**3**	Pd(II)	1.59	71
	Zn(II)	3.15	55
	Ni(II)	3.62	22
**4**	Pd(II)	1.40	74
	Zn(II)	2.62	58
	Ni(II)	3.44	25
**5**	Pd(II)	1.26	77
	Zn(II)	2.33	60
	Ni(II)	3.25	27
**6**	Pd(II)	1.14	80
	Zn(II)	1.90	63
	Ni(II)	2.87	29
**7**	Pd(II)	1.05	82
	Zn(II)	1.64	66
	Ni(II)	2.75	32
**8**	Pd(II)	0.95	86
	Zn(II)	1.38	70
	Ni(II)	2.65	36
**9**	Pd(II)	0.80	89
	Zn(II)	1.10	74
	Ni(II)	2.57	42

**Table 4:** Comparison of the stability constants (β_n_) and partition constants (P_n_) of M(II) complexes with 1,2,4-triazole and 1-alkyl-1,2,4-triazole and at 20 °C.

Ligand	Metal Ion	Stability Constants, β_n_		Partition Constants P_n_	
		log β_1_	log β_2_	log P_1_	log P_2_
1,2,4-triazole	Ni(II) [23]	5.96	10.54	4.81	6.17
	Zn(II) [23]	5.03	8.8	3.79	5.86
1-pentyl-1,2,4-triazole	Pd(II)	4.72	9.32	2.46	5.23
**1**	Ni(II)	2.15	5.11	1.05	2.74
	Zn(II)	3.42	7.43	1.88	3.56
1-hexyl-1,2,4-triazole	Pd(II)	4.78	9.43	2.54	5.41
**2**	Ni(II)	2.26	5.37	1.09	2.84
	Zn(II)	3.5	7.6	1.9	3.6
1-heptyl-1,2,4-triazole	Pd(II)	4.83	9.52	2.61	5.53
**3**	Ni(II)	2.29	5.44	1.12	2.92
	Zn(II)	3.55	7.71	2.06	3.9
1- octyl-1,2,4-triazole	Pd(II)	4.89	9.64	2.76	5.96
**4**	Ni(II)	2.35	5.58	1.14	2.97
	Zn(II)	3.58	7.78	2.13	4.03
1-nonyl-1,2,4-triazole	Pd(II)	4.92	9.71	2.82	6.09
**5**	Ni(II)	2.38	5.66	1.18	3.08
	Zn(II)	3.61	7.84	2.19	4.15
1-decyl-1,2,4-triazole	Pd(II)	4.95	9.76	2.88	6.22
**6**	Ni(II)	2.41	5.72	1.21	3.15
	Zn(II)	3.69	8.02	2.24	4.24
1-dodecyl-1,2,4-triazole	Pd(II)	5.01	9.88	2.95	6.31
**7**	Ni(II)	2.52	6	1.28	3.34
	Zn(II)	3.74	8.13	2.27	4.3
1-tetradecyl-1,2,4-triazole	Pd(II)	5.06	9.98	3.12	6.37
**8**	Ni(II)	2.63	6.25	1.32	3.44
	Zn(II)	3.88	8.43	2.35	4.45
1-hexacedyl-1,2,4-triazole	Pd(II)	5.12	10.1	3.39	6.92
**9**	Ni(II)	2.67	6.35	1.32	3.48
	Zn(II)	3.94	8.56	2.38	4.51

**Table 5:** The initial fluxes for competitive transport of Pd(II), Zn(II) and Ni(II) ions across PIMs doped with 1-pentyl-1,2,4-triazole (**1**) and recovery factor after 24 h; membrane: 2.6 cm^3^ *o*-NPPE/1 g CTA; feed phase: [M^2+^] = 0.001 mol/dm^3^ each metal ion, receiving phase: 0.1 mol/dm^3^ HCl.

Concentration of Carrier, mol/dm^3^	Metal Ions	Initial Flux, J_0_ µmol/m^2^∙s	RF after 24 h, %
**0.25**	Pd(II)	0.35	40.5
	Zn(II)	0.04	5.6
	Ni(II)	0.01	1.3
**0.50**	Pd(II)	2.95	94.7
	Zn(II)	0.73	38.6
	Ni(II)	0.22	14.5
**1.00**	Pd(II)	2.65	89.3
	Zn(II)	0.85	44.4
	Ni(II)	0.25	18.1
**1.50**	Pd(II)	2.41	78.2
	Zn(II)	1.08	47.3
	Ni(II)	0.34	28.5

**Table 6:** The roughness and average thickness for PIM with 1-alkyl-1,2,4-triazoles.

	Polymer Inclusion Membranes with Alkyl-Triazole (1–9)								
**Carrier**	**1**	**2**	**3**	**4**	**5**	**6**	**7**	**8**	**9**
**Roughness**, nm	4.23	4.71	5.01	5.25	6.30	8.39	8.41	8.56	8.82
**Average thickness**, μm	30	29	32	29	32	33	31	32	33

**Table 7:** Influence of chloride ion concentration in the feed phase on kinetic parameters, order and Pd(II) ion separation coefficient in transport across PIMs with 1-pentyl-1,2,4-triazole (**1**). Membrane: 2.6 cm^3^ o-NPPE/1g CTA, 0.5 mol/dm^3^ **1** calculation on the plasticizer’s volume; feed phase: [M^2+^] = 0.001 mol/dm^3^ each metal ion, receiving phase: 0.1 mol/dm^3^ HCl.

Concentration of Chloride Ions, mol/dm^3^	Metal Ions	Initial Flux, J_0_ µmol/m^2^∙s	Order and Separation Coefficient S_Pd(II)/M(II)_
**-**	Pd(II)	2.95	Pd(II) > Zn(II) > Ni(II) 4.0 13.4
	Zn(II)	0.73	
	Ni(II)	0.22	
**0.5**	Pd(II)	5.78	Pd(II) > Zn(II) > Ni(II) 2.2 5.4
	Zn(II)	2.64	
	Ni(II)	1.02	
**1.0**	Pd(II)	7.67	Pd(II) > Zn(II) > Ni(II) 1.5 6.1
	Zn(II)	5.19	
	Ni(II)	1.26	

**Table 8:** The initial fluxes, selectivity order and selectivity coefficients for competitive transport of Pd(II), Zn(II), and Ni(II) ions across PIMs doped with 1-alkyl-1,2,4-triazole (**1**–**9**), membrane: 2.6 cm^3^ *o*-NPPE/1g CTA, 0.5 mol/dm^3^ **1**–**9** calculation on the plasticizer’s volume; feed phase: [M^2+^] = 0.001 mol/dm^3^ each metal ion, receiving phase: 0.1 mol/dm^3^ HCl.

Carrier	Metal Ions	J_0_, μmol/m^2^·s	Selectivity Coefficient S_Pd(II)/M(II)_
**1**	Pd(II)	2.95	Pd(II) > Zn(II) > Ni(II) 4.0 13.4
	Zn(II)	0.73	
	Ni(II)	0.22	
**2**	Pd(II)	3.17	Pd(II) > Zn(II) > Ni(II) 3.5 11.7
	Zn(II)	0.91	
	Ni(II)	0.27	
**3**	Pd(II)	3.58	Pd(II) > Zn(II) > Ni(II) 3.2 10.5
	Zn(II)	1.12	
	Ni(II)	0.34	
**4**	Pd(II)	3.75	Pd(II) > Zn(II) > Ni(II) 2.9 9.9
	Zn(II)	1.31	
	Ni(II)	0.38	
**5**	Pd(II)	4.09	Pd(II) > Zn(II) > Ni(II) 2.6 9.3
	Zn(II)	1.56	
	Ni(II)	0.44	
**6**	Pd(II)	4.43	Pd(II) > Zn(II) > Ni(II) 2.5 8.7
	Zn(II)	1.77	
	Ni(II)	0.51	
**7**	Pd(II)	4.68	Pd(II) > Zn(II) > Ni(II) 2.3 8.2
	Zn(II)	2.05	
	Ni(II)	0.57	
**8**	Pd(II)	4.92	Pd(II) > Zn(II) > Ni(II) 2.0 7.5
	Zn(II)	2.47	
	Ni(II)	0.66	
**9**	Pd(II)	5.36	Pd(II) > Zn(II) > Ni(II) 1.9 6.5
	Zn(II)	2.81	
	Ni(II)	0.82	

**Table 9:** Diffusion coefficients normalized for competitive transport of Pd(II), Zn(II) and Ni(II) ions through PIM doped with 1-pentyl-triazole (**1**) and 1-hexadecyl-triazole (**9**).

Carrier	Metal Ion	Δ_o_, s/m	D_o_, cm^2^/s	D_o,n_, cm^2^/s
**1**	Pd(II)	124.08	2.38 × 10^−7^	4.53 × 10^−8^
	Zn(II)	195.83	4.21 × 10^−8^	8.02 × 10^−9^
	Ni(II)	1416.27	1.47 × 10^−11^	2.80 × 10^−12^
**9**	Pd(II)	136.49	2.64 × 10^−7^	6.31 × 10^−8^
	Zn(II)	204.16	5.96 × 10^−8^	1.42 × 10^−8^
	Ni(II)	1483.21	2.02 × 10^−11^	4.83 × 10^−12^

## Data Availability

The data presented in this study are available on request from the corresponding author.
